# Public health round-up

**DOI:** 10.2471/BLT.24.010924

**Published:** 2024-09-01

**Mpox emergency**

A health official takes a sample from an mpox patient in the Democratic Republic of the Congo which is at the epicentre of an mpox outbreak declared to be a public health emergency of international concern by the World Health Organization on 14 August.

**Figure:**
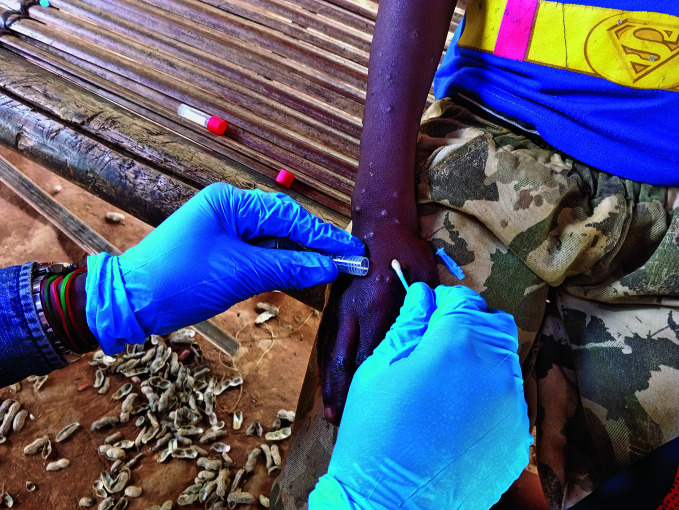
WHO/Katson Maliro

## Mpox emergency

World Health Organization (WHO) Director-General Tedros Adhanom Ghebreyesus declared that the upsurge of mpox in the Democratic Republic of the Congo (DRC) and a growing number of countries in the African region constitutes a public health emergency of international concern (PHEIC) under the International Health Regulations (2005) (IHR).

The 14 August declaration was made on the advice of an IHR Emergency Committee of independent experts who reviewed data presented by WHO and affected countries. 

In declaring the PHEIC, Dr Tedros said, “The emergence of a new clade of mpox, its rapid spread in eastern DRC, and the reporting of cases in several neighbouring countries are very worrying. On top of outbreaks of other mpox clades in DRC and other countries in Africa, it’s clear that a coordinated international response is needed to stop these outbreaks and save lives.”

WHO is coordinating a multifaceted response with local governments, the Africa Centers for Disease Control and Prevention and other partners, emphasizing community engagement and the avoidance of travel bans. A 15 million United States dollars (US$) regional plan is supporting surveillance and response, bolstered by US$ 1 million from the WHO Contingency Fund for Emergencies.

The Director-General triggered the process for Emergency Use Listing (EUL) the two vaccines for mpox that have been approved by WHO-listed national regulatory authorities, and which are recommended by WHO’s Strategic Advisory Group of Experts on Immunization.

The EUL will accelerate vaccine access, notably by enabling partners including Gavi, the Vaccine Alliance and the United Nations Children’s Fund (UNICEF) to procure vaccines for distribution.

https://bit.ly/3YHNAlh

https://bit.ly/3SLUfHg

https://bit.ly/4dnGnvc

## Polio in Gaza

Type 2 circulating vaccine-derived poliovirus (cVDPV) was detected in Gaza’s wastewater in June. Variant strains of the virus were detected in six wastewater samples, collected from two environmental surveillance sites in Khan Younis and Deir al Balah.

According to a 16 July statement by the Global Polio Eradication Initiative, genomic sequencing of the isolates indicates that the strains are closely related to each other and to a cVDPV variant that was circulating in Egypt during the second half of 2023. Genetic changes in the isolates suggest that they may have been introduced into Gaza as early as September 2023.

While there have been no reports of poliovirus disease in Gaza, there is serious concern about the risk of unvaccinated children being infected, notably infants who may not have been vaccinated over the nine months of conflict.

In a 7 August media briefing, Director-General Tedros stated that WHO was sending more than 1 million polio vaccines to Gaza, with the intention of rolling them out in the following weeks. He added that WHO is also supporting routine immunization and disease surveillance, including for polio. He called for a ceasefire or, at the very least, “days of tranquility” during the preparation and delivery of the vaccination campaigns.

In related news, the 39th Emergency Committee meeting on the international spread of poliovirus unanimously agreed that the risk of international spread of poliovirus remains a PHEIC. The committee met on 8 July to review data on wild poliovirus (WPV1) and cVDPV, and, according to a 13 August statement, recommended the extension of the PHEIC in view of the renewed spread of WPV1 in formerly endemic areas and core reservoirs of Afghanistan and Pakistan; and the re-establishment of WPV1 transmission in Kandahar (Afghanistan) and Peshawar (Pakistan).

https://bit.ly/46LYOHA

https://bit.ly/46G3Vcp

https://bit.ly/4djmi9B

## Global hunger setbacks

Around 733 million people faced hunger in 2023, equivalent to one in eleven people globally and one in five in Africa.

This is according to the latest *State of Food Security and Nutrition in the World* report published on 24 July by five United Nations specialized agencies. Launched this year in the context of the G20 Global Alliance against Hunger and Poverty Task Force Ministerial Meeting in Brazil, the report warns that the world is falling significantly short of achieving Sustainable Development Goal (SDG) 2, Zero Hunger, by 2030.

The report shows that, despite progress in specific areas such as stunting and exclusive breastfeeding, the world has been set back 15 years, with levels of undernourishment comparable to those in 2008-2009.

https://bit.ly/3YLDSP8

## Gambia maintains ban on female genital mutilation

Gambia’s parliament voted by a substantial margin on 15 July to uphold a ban on female genital mutilation (FGM), defeating a bill put forward by pro-FGM, traditionalist lawmakers and religious leaders.

The decision to maintain the FGM ban aligns with Gambia’s international and regional commitments to prevent harmful practices against girls and women, consistent with the Convention on the Rights of the Child, the Convention on the Elimination of All Forms of Discrimination Against Women, the African Charter on the Rights and Welfare of the Child, and the Maputo Protocol protecting African women’s rights.

A joint statement issued by UNICEF and partner agencies commended the decision, stressing the vital importance of keeping Gambia’s legal protections in place.

https://bit.ly/3SRrfxV

## Violence against adolescent girls

Nearly a quarter of adolescent girls in relationships – close to 19 million – will experience physical and/or sexual intimate partner violence by their 20th birthday.

This is according to a study published in *The Lancet Child & Adolescent Health* on 29 July. The study uses data from WHO’s global database on the prevalence of violence against women and employs Bayesian hierarchical modelling methods to generate internationally comparable estimates.

The study examines both the lifetime and past 12-month prevalence of physical and/or sexual intimate partner violence against girls aged 15–19. Other forms of violence, such as psychological violence, are not included due to the lack of an internationally comparable measure.

While there are significant differences in prevalence between countries, currently no country is on track to eliminate violence against women and girls by the 2030 sustainable development goal target date.

https://bit.ly/3AlxUKp

## Research to prepare for the next pandemic

The Coalition for Epidemic Preparedness Innovations (CEPI) and WHO called on researchers and governments to strengthen and accelerate global research to prepare for the next pandemic, notably by widening the scope of pathogen research.

The appeal, issued during the Global Pandemic Preparedness Summit held in Rio de Janeiro, Brazil, on 29 and 30 July, emphasized the need to expand research to include entire families of pathogens that can infect humans, regardless of their perceived pandemic risk. The proposed approach involves using prototype pathogens as guides to develop a broader knowledge base for entire pathogen families.

"WHO’s scientific framework for epidemic and pandemic research preparedness is a vital shift in how the world approaches countermeasure development, and one that is strongly supported by CEPI," said Dr Richard Hatchett, CEPI’s chief executive officer.

https://bit.ly/4dl85J0

**Cover photo**

Children at the therapeutic feeding centre in the Harf Sufyan district of the Amran governorate in Yemen.

**Figure:**
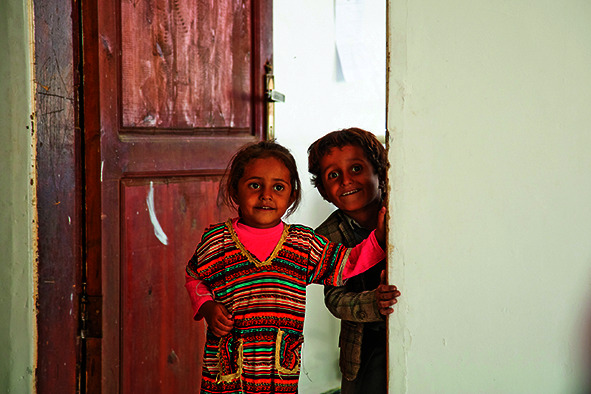
WHO/Gabreez

## Accelerating the development of H5N1 vaccines

A new project aiming to accelerate the development and accessibility of human avian influenza (H5N1) messenger RNA (mRNA) vaccine candidates for manufacturers in low- and middle-income countries was launched on 29 July.

The project will be led by Argentinian manufacturer Sinergium Biotech with the support of WHO and the Medicines Patent Pool (MPP) mRNA Technology Transfer Programme.

Sinergium Biotech has developed candidate H5N1 vaccines and is working to establish proof-of-concept in preclinical models. If successful, the company will share data, technology, materials, and expertise with other manufacturing partners.

https://bit.ly/4crg5ah

## Guidance on human strongyloidiasis

WHO released guidance on human strongyloidiasis on 2 August. Caused by the helminth *Strongyloides stercoralis*, human strongyloidiasis is a parasitic disease affecting an estimated 300–600 million people globally, particularly in tropical regions. It is associated with a range of clinical presentations, including diarrhoea, abdominal pain and urticaria.

The guidance recommends annual mass drug administration with single-dose ivermectin for all individuals aged 5 years and above in endemic areas where the prevalence among school-aged children is 5% or higher. The guidance also provides details on implementation and highlights the need for further research to refine public health strategies for strongyloidiasis.

https://bit.ly/3WKQfIh

**Looking ahead**

2–4 September. 15th World Conference on Injury Prevention and Safety Promotion. New Delhi, India. https://bit.ly/3Wnh06W

20–23 September. World One Health Congress. Cape Town, South Africa https://globalohc.org/

26 September. United Nations General Assembly High-Level Meeting on antimicrobial resistance 2024. New York, United States of America. https://bit.ly/4cnpfVf

